# Progress in Fevers

**Published:** 1901-04-20

**Authors:** 


### PROGRESS IN FEVERS.

(*Continued from page* 27.)

**Yellow Fever.**—As long ago as 1882 [1] Finlay and Gerhard concluded that the mosquito conveys the infection of yellow fever. Finlay regarded the mosquito not as an intermediate host, but merely as the transmitter of the disease to man. The germ might also be transmitted to the next generation of mosquitoes. He recalls [2] how in a paper written in 1891 he advocated the mosquito theory of propagation, and sought to prove his point by reference to the Saint-Nazaire epidemic of 1861. The severity of the attack he considers dependent on the number of bites. Yellow fever flourishes at low altitudes, near the sea coast, in hot countries; conditions also favourable to mosquito life. These insects become benumbed at temperatures below 60° Fahr. But yellow fever also flourishes where filth and defective sanitation prevail, as pointed out recently by A. H. Doty and Turnbull, and its presence is not always coincident with the existence of mosquitoes in abundance, as shown by several United

States epidemics.[3] The conclusions arrived at by W. Reed, J. Carroll, and Agramonte, of the United States Army Commission,[4] as a result of their earliest investigations, viz. that the bacillus icteroides (bacillus of Sanarelli) was either absent or only accidentally present in yellow fever and stood in no causative relation to it; and that the mosquito served as the intermediate host of the parasite of yellow fever, and propagated the disease by its bite, were based on insufficient grounds. The conditions of these experiments, by which unfortunately Dr. Lazear lost his life, were not sufficiently rigid to justify the conclusions, although they cast discredit on the bacillus of Sanarelli.[5] This bacillus is now proved[6] to be identical in behaviour and reactions with the hog cholera bacillus, while the bacillus *x* of Sternberg, which he claimed was identical with B. icteroides, is now placed among the coli group.

Later experiments, by Reed and his colleagues,[7] announce a completely successful result in 80 per cent. of their mosquito experiments. These were all conducted on Spanish immigrants wishful for immunity and willing to take the risks. Strict quarantine and exclusion of other sources of infection was observed. The experiments were conducted on human beings, because animals are insusceptible to the disease. The following[8] are some of the conclusions reached: The musquito, *Culex fasciatus* (said by Howard to be identical with *Culex tiniado* of Giles), acts as the intermediate host for the parasite of yellow fever. The mosquito, contaminated by biting a yellow-fever patient, can after twelve days (during which the parasite is developing in its body) transmit the disease by biting a non-immunised individual. Subcutaneous injection of the blood of a stricken individual also induces the disease. The mosquito *propagates* the disease; the specific cause remains to be discovered. Fomites and houses, apart from the presence of contaminated mosquitoes, do not spread the disease. Susceptible individuals using for weeks the bedding and garments of infected patients remain free.[9]

Klebs, of Chicago, has described oval amœboid bodies which he isolated from the liver and other organs of infected patients.[10] H. E. Durham and the late W. Meyers[11] have quite recently found in the organs of all fatal cases of yellow fever examined a fine small influenza-like bacillus present in almost "pure culture" in the "mucus" of yellow-fever stools, not numerous elsewhere. Sternberg has mentioned it. It stains very slowly and with great difficulty, and cannot be grown in ordinary culture tubes. Careful search for protozoal parasites failed to find such as a cause of the disease. The mosquito theory of propagation hardly agrees with this theory of origin. "The acquisition of a new intestinal bacterium would explain the immunity of the 'acclimatised.'"

**Tetanus.**—Attention directed to the probable microbic origin of tetanus resulted in the finding of a specific bacillus by Nicolaier and Rosenbach about 1885.[12] Since the bacillus remains confined to the vicinity of inoculation the symptoms must be due to absorption of the toxins produced. Subcutaneous injection of antitoxin was tried in 1891 without much reduction in mortality. In 1898 injection into the brain or subdural space was tried, since by subcutaneous injection only the toxin in the blood was neutralised, not that already firmly combined with the motor nerve-cells. At least one case of fatal cerebral abscess has followed. The best line of treatment at present seems to be thorough and early removal of the source of infection, and early injection of antitoxin, before the poison in the blood has combined with the nerve cells. Sedatives are valuable for controlling symptoms and carbolic acid internally deserves further trial. Woodruff[13] strongly recommends large doses of Calabar bean and chloral. Moscheowitz[14] considers rational treatment includes five important points:—destruction of bacteria at the seat of infection; elimination of toxins already absorbed (venesection and normal saline injection are tentatively suggested); neutralisation of toxins present (antitoxin has reduced the mortality from 90 to 40 per cent.); prophylactic injection where tetanus is foreseen; use of nerve sedatives. Krokiewicz[15] has treated two very severe cases by subcutaneous injection of an unfiltered emulsion of rabbit's brain: one case recovered and probably the other would have recovered but for an accident. Of ten cases so treated eight recovered—one after intracerebral injection of antitoxin had failed. Wilms[16] has found antitoxin useless in acute cases. Milder cases he thinks would have recovered as well without it. Thalmann,[17] experimenting on guinea-pigs, finds the intestine and bladder insusceptible to infection, whether healthy or not. Infection by inhalation is common if any catarrh or slight abrasion of the respiratory tract is present.

S. H. Long[18] advocates injection of large doses which may obviate the necessity for intracranial injections. He gave 680 c.cm. of antitoxin in fourteen days to a boy of thirteen who developed symptoms four days after injury: 10 c.cm. were injected every four hours for thirteen doses, starting on the seventh day from the injury; the rest was given per rectum. Transient urticaria was observed. Practically no drugs were given. The serum cost £10 10s. Woodroffe[19] injected 501 c.cm. into a girl of eleven who developed moderate symptoms ten days after injury. Urticaria was noticed. Giles[20] obtained recovery by pushing potassium bromide in a boy of thirteen, who showed symptoms in thirteen days. One dose of antitoxin (20 c.cm.) was used. Heckel and Reyné consider, he says, that in such forms as puerperal tetanus the serum is much less efficacious. Lyster[21] also got good result with antitoxin. In one case,[22] occurring ten days after head injury, Victor Horsley excised the sloughing wound and injected 7½ c.cm. of serum subdurally. 20 c.cm. were also injected into the flank, and chloral up to 80 grains daily given throughout. Improvement began in a week. Fourteen deaths and eleven recoveries are reported out of twenty-five cases of intracranial injection. E. C. Hayes[23] reports a fatal case occurring in military practice in India. Symptoms followed in ten days after a severe burn of the hand. Chloral and later morphia in full doses were given, also carbolic acid, five minims of a one per cent. solution, hypodermically. Locally a bath of corrosive sublimate and tartaric acid was tried. On the second day 1½ grams of dried antitoxic serum were injected and repeated next day. Death occurred somewhat unexpectedly the same day. *Post-mortem* marked congestion of the brain and cord was found, especially of the superficial veins. The nerves exposed by the injury were not congested.

[1] Med. Rec., Nov. 3, p. 697. [2] Ibid., Jan. 19. [3] Ibid., Nov. 3. [4] Ibid. [5] Brit. Med. Jour., Nov. 10. [6] Brit. Med. Jour., Jan. 19, p. 169, [7] Brit. Med. Jour., Jan. 12, p. 110. [8] Med. Rec., Feb. 16. [9] Brit. Med. Jour., Feb. 16, p. 417. [10] Med. Rec., Nov. 24. [11] Brit. Med. Jour., Feb. 23. [12] Lancet. Nov. 7, p. 1440. [13] Columbus Med. Jour., Dec. [14] Brit. Med. Jour., Nov. 24. [15] Brit. Med. Jour., Dec. 15. [16] Brit. Med. Jour., March 2. [17] Brit. Med. Jour., Feb. 2. [18] Brit. Med. Jour., Nov. 24. [19] Brit. Med. Jour., Feb. 16. [20] Aust. Med. Gaz., Nov. 20. [21] Brit. Med. Jour., Feb. 9. [22] Lancet, Nov. 17. p. 1420. [23] Brit. Med. Jour., Dec. 22.

